# Latissimus Dorsi Myocutaneous Flap for Breast Reconstruction: Bad Rap or Good Flap?

**Published:** 2011-10-17

**Authors:** Galen Perdikis, Stephanie Koonce, George Collis, Dustin Eck

**Affiliations:** Mayo Clinic, Jacksonville, FL

## Abstract

**Objective:** This article serves to review latissimus dorsi myocutaneous flap as an option for breast reconstruction postmastectomy. Since the introduction of the latissimus dorsi myocutaneous flap in the late 1970s, its use has always been as a secondary technique, particularly after the development of the transverse rectus abdominus myocutaneous flap in the 1980s. **Methods:** A literature review of the history of latissimus dorsi myocutaneous flap utilized for breast reconstruction as well as a review of our institution's experience with latissimus dorsi myocutaneous flap and tissue expander placement was performed. **Results:** There remains a paucity of published studies investigating latissimus dorsi myocutaneous flap for breast reconstruction. Most studies have small numbers and do not utilize tissue expanders. More recently several small studies have been published that show acceptably low complication rates with aesthetically pleasing outcomes when latissimus dorsi myocutaneous flap is employed with a tissue expander. At our institution, we have employed latissimus dorsi myocutaneous flap with tissue expander placement for both delayed and immediate reconstruction with subsequent replacement with a permanent implant with a capsular contraction rate of 10.5%. Our data and others more recently published demonstrate very acceptable capsular contracture rates and aesthetic outcomes, particularly when an expander is utilized. **Conclusion:** The latissimus dorsi myocutaneous flap remains an excellent choice for breast reconstruction with a low risk of complications.

Over 202,000 women were diagnosed with breast cancer in 2007.^1^ As the US population continues to age, the prevalence of breast cancer is expected to continue to increase. The choice to undergo breast reconstruction is increasingly commonplace and has proven psychological benefits for many women.^2^ The type and the timing of reconstruction is a multifactorial decision based on the need for adjuvant treatment, lifestyle, desired cosmetic outcome, and preference and experience of the surgeon. As reconstruction has become more prevalent, the search for the most aesthetically pleasing outcome has become more pronounced. Over the past several decades breast reconstruction techniques have evolved from injection of paraffin directly into the defect to the current advanced techniques such as deep inferior epigastric perforator flaps.^3^ This article discusses the history of the latissimus dorsi myocutaneous flap (LDMF), its evolution and reviews its published data.

## METHODS

A literature review of the history of LDMF utilized for breast reconstruction as well as a review of our institution's experience with LDMF and tissue expander placement was performed.

## RESULTS

The LDMF was first described in the late 1800s by Italian surgeon Tanzini^4^ as a novel approach to repairing breast amputation. It was not until the 1970s however that the LDMF began to evolve into its current state. Schneider et al^5^ and Olivari^6^ described their experiences with latissimus flaps following mastectomy and radiation in 1977. In 1978, the technique of a skin island over the muscle flap was promoted by Bostwick et al.^7^ Bostwick et al^7^^,^^8^ described utilization of the LDMF with and without silicone prosthesis. Later, Maxwell argued that the flap could be utilized even if surgeons sacrificed the main thoracodorsal trunk as the flap could be sustained by collateral circulation.^9^ This collateral circulation was found by Fisher to be from reversed flow of the serratus branch of the thoracodorsal artery.^10^

In early series, LDMF was heavily criticized for high capsular contracture rates and other complications such as seroma formation.^11^^-^14 Also, in the early 1980s, Hartrampf et al^15^ popularized the transverse rectus abdominus myocutaneous (TRAM) flap. The TRAM flap quickly became the first choice for many patients considering breast reconstruction, even though it was a more extensive surgery with a longer recovery period and had significant complications. With this rise in the popularity of TRAM flaps, together with the bad rap for high capsular contracture rates, LDMF was relegated to a secondary choice.

As stated, the most significant criticism has been the LDMF's reported incidence of capsular contracture (7.4%-75%).^11^^-^14 The majority of the data on LDMF complication rates however are based on small series that are both antiquated and biased.^12^^-^14 Furthermore, upon review of the published series, the majority of papers utilized an implant with the flap as the definitive reconstruction rather than an expander. For example, DeMey et al^13^ reviewed 103 cases of LDMF with permanent implant placement. They noted clinically significant capsular contractures in 26% of patients. Again, no tissue expanders were utilized prior to permanent implant. Kroll et al^14^ compared LDMF (n = 16) to TRAM (n = 66) in previously irradiated patients. There was a much higher complication rate in the LDMF group (63% versus 33%). Again, no patients in this study had tissue expanders placed prior to permanent implant placement.

McCraw and Maxwell^12^ reported the results of 82 patients who had undergone LDMF for breast reconstruction. Permanent implants were used in all patients. The patients were split into 2 groups—radical mastectomy and modified radical mastectomy. A capsular contracture rate of 75% was noted in the radical mastectomy group and of 39% in the modified radical mastectomy group.^12^ Corrective surgery was required in 44 and 40 cases, respectively.^12^ The authors noted that tissue expander placement would have perhaps decreased their capsular contracture rate.

More recently, however, Venus and Prinsloo^11^ reported a small series (n = 38) of immediate latissimus dorsi plus permanent implant reconstructions in 2008. Their survey focused primarily on patient satisfaction at a mean follow-up time of 3.2 years, but they did note a low capsular contracture rate requiring surgery of 7.4% and a seroma rate of 20.4%.^11^ Also in 2008, Hankins and Friedman^16^ reported a small series of LDMF plus permanent implant reconstruction that had a total complication rate of 27% with 2 of their 37 patients developing capsular contracture.

There continues to be a dearth of literature investigating LDMF breast reconstruction in conjunction with initial placement of tissue expanders rather than an implant. Abdalla et al^17^ published a series of 25 women who underwent immediate LDMF reconstruction with tissue expander placement after skin sparing mastectomy.^17^ They reported skin flap necrosis in 12% and wound infection in 4%; no mention was made of capsular contracture. Mast and Simoneau^18^ describe a capsular contracture rate of 8% in 32 patients with LDMF reconstruction with tissue expander and subsequent permanent implant placement. Another small series of 32 consecutive patients from Sweden investigated patient satisfaction with LDMF flap alone (n = 8), with tissue expanders (n = 13), and with permanent implants (n = 11).^19^ Their median follow-up time was 35 months, and they noted 9 of 26 patients developed seromas without mention of capsular contracture rates.^19^ Patients overall were satisfied with aesthetic appearance.^19^

There is variation in published complication rates in LDMF in irradiated breasts as well. Garusi et al^20^ reported a 3.1% Baker's Class III capsular contracture rate in 63 patients who had LDMF with permanent implant reconstruction after irradiation. Spear et al^21^ conducted a retrospective review of 28 patients after irradiation, 18 of whom had LDMF with tissue expander and 10 of whom had LDMF with permanent implant.^21^ They reported a capsular contracture rate of 3.5% and a seroma rate of 17.8% (5 of 28 patients).^21^ Chang et al^22^ published an extensive review of 1000 reconstructions of previously irradiated breasts with various reconstructive methods. They reported a total capsular contracture rate for LDMF of 6.4% in breasts that did not receive radiation and 3% in those that received preoperative radiation.^22^ However, the LDMF flaps they studied also employed permanent implants rather than tissue expanders. All in all, the data remain clouded by consisting mainly of small series.

## DISCUSSION

**Figure 1:**
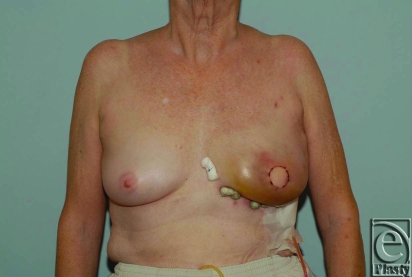
A patient a few days after skin sparing mastectomy and immediate breast reconstruction. The bolsters are utilized for inset of the latissimus dorsi muscle in the inframammary fold.

At our institution, we have employed LDMF with tissue expander placement for both delayed and immediate reconstruction with subsequent replacement with a permanent implant (Fig 1). We published a review of our experience with 100 patients who underwent LDMF with tissue expanders and demonstrated a capsular contracture rate of 6%.^23^ We also published our experience with LDMF following mastectomy for failed lumpectomy and radiation. Even in this irradiated group, when an expander is used, the capsular contracture rate is 12%.^24^ More recently, our review of more than 200 patients including radiated and nonradiated breasts confirms a low capsular contracture rate of any grade of 10.5%. Seroma, both donor site and breast, remains significant (24.5%). This rate remains consistent with previously published studies.^13^

**Figure 2:**
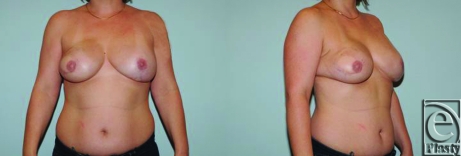
A patient after delayed reconstruction of right breast with latissimus dorsi myocutaneous flap.

**Figure 3:**
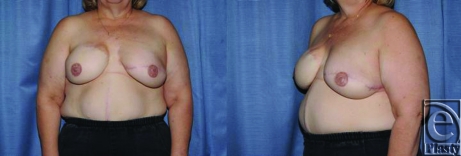
A patient following reconstruction of the left breast with latissimus dorsi myocutaneous flap and expander following a failed deep inferior epigastric perforator flap (DIEP) flap. The right side was reconstructed with a DIEP flap.

Aesthetically, a pleasing outcome is obtainable with LDMF reconstruction when tissue expanders are employed (Figs 2 and 3). The advantage of this operation is that it is technically straightforward and hospitalization is an average of 2 days, which compares favorably with the TRAM flap. Transverse rectus abdominus myocutaneous also has an arguably higher rate of both overall complications and significant complications such as hernias, fat necrosis, etc.^25^^-^28

In summary, LDMF for breast reconstruction after mastectomy is a procedure that has been erroneously maligned over the years for high capsular contracture complications. In reality, a lot of the data regarding capsular contracture rates in LDMF are outdated and skewed. Proper study of LDMF most likely was hindered by the rapid rise in the popularity of the TRAM flap in the early 1980s. Our data and others more recently published demonstrate very acceptable capsular contracture rates and aesthetic outcomes, particularly when an expander is utilized. We believe that LDMF should be utilized more often, as it is a technically straightforward procedure that gives acceptable cosmetic outcomes with few complications.
